# Development and Pilot Testing of an Internet-Based Self-Help Intervention for Depression for Indian Users

**DOI:** 10.3390/bs8040036

**Published:** 2018-03-22

**Authors:** Seema Mehrotra, Paulomi Sudhir, Girish Rao, Jagadisha Thirthalli, TK Srikanth

**Affiliations:** 1 Department of Clinical Psychology, National Institute of Mental Health and Neurosciences, Bangalore, Karnataka 560029, India; paulomi@nimhans.ac.in; 2 Department of Epidemiology, Center for Public health, NIMHANS, Bangalore 560029, India; girishnrao@gmail.com; 3 Department of Psychiatry, NIMHANS, Bangalore 560029, India; jagatth@yahoo.com; 4 E-health Research Center, IIIT, Bangalore 560100, India; tk.srikanth@iiitb.ac.in

**Keywords:** internet-based self-help, depression, treatment gap, depression app, guided self-help, depression in India

## Abstract

There is a dearth of published research on uptake and utility of mental health apps in India, despite a rising global trend in the application of technology in the field of mental health. We describe the development and pilot testing of a self-help intervention for depression, PUSH-D (Practice and Use Self-Help for Depression) for urban Indians. This guided self-help app, with essential and optional zone sections, was developed to provide a comprehensive coverage of therapeutic strategies drawn from cognitive behavior therapy, interpersonal therapy, supportive psychotherapy, and positive psychology. Pilot testing was carried out using a single group pre-, post- and follow-up design in 78 eligible participants. Participants were typically young adults with major depression or dysthymia and significant impairment in functioning. Almost two-thirds of the participants had never sought professional mental health help. Significant reductions in depression and improvement in the functioning and well-being were notedon standardized measures in participants completing all 10 essential zone sections. These gains were maintained at follow-up. The results were similar for partial completers, who completed fiveout of the 10 essential sections. PUSH-D is one of the first indigenously developed self-help apps for depression and it shows promise in reducing the treatment gap for depression in India.

## 1. Introduction

The potential for the use of internet-based platforms in the mental health field has attracted significant attention of scientists and other stakeholders in the last few years [1,2,3]. This is evident from the number of articles being published on this topic as well as the number of applications (apps) available on platforms such as Apple and Google Play stores [4]. Using the key word ‘mental health’ in combination with the word ‘internet-based’ as search terms on PubMed resulted in 251 articles for the last five-year period(2013–2017).

A growing number of research studies on internet-based interventions in the field of mental health need to be understood in the background of observations that the majority of individuals across the globe who need mental health interventions do not receive them. World Health Organization (WHO, 2007) has recommended development of an optimal mix of different kinds of services for addressing treatment gap in mental health. This includes empowerment of communities, through provision of low-intensity self-help interventions as a first line in themanagement of common mental health problems [5,6]. Realization of the magnitude of treatment gap and the potential use of technology on one hand, coupled with increasing internet access on the other hand have propelled voluminous research on self-help interventions for psychiatric conditions that can be delivered through the internet [7]. Internet-based self-help interventions can play an important role in addressing unmet mental health needs in low-resource settings with limited availability of mental health professionals and poor access to mental health services. Internet-based interventions offer several advantages such as ease of access, low costs, flexibility, privacy and convenience of use etc. [1]. Internet-based self-help interventions have several potential points of implementation: during the waiting period for meeting a mental health professional, as stepped-care for low severity problems, as preventive measure for individuals at risk, as relapse prevention for those with residual symptoms or as a blended approach when integrated with face-to-face interventions [1]. The present study focuses on examining an internet-based self-help intervention, primarily as a stepped-care approach.

## 2. Literature Review

### 2.1. Current Status at a Glance

Numerous studies have been published on internet-based interventions for a wide variety of common mental health conditions and substance use disorders [3]. Cognitive behavior therapies(CBT) , especially for depression, have been the most popular forms of therapy approaches for which internet-based formats have been tested, although there are a handful of studies on other forms of internet-based therapies too [1,8,9]. This may be due to a variety of factors such as availability of empirical evidence-base for CBT as well as its structured nature, ease of transformation into internet-based delivery formats and potential for client-learning with minimal external assistance. CBT-based internet interventions have typically yielded moderate effect sizes [10]. Internet-based interventions that are completely unguided, as well as those involving some level of human assistance have both been examined for their utility. Most studies suggest superiority of interventions that involve some human contact (therapist or a non-professional) in terms of adherence and performance [11]. Unguided self-care apps tend to be associated with low adherence rates. Typically, unguided apps also have limited scope for contextualization depending on the needs of a user. Other limitations of completely unguided apps include dependence on a user’s motivation to complete the intervention and reliance on a user’s self-awareness about when it is required to stepup to a higher-intensity intervention. There is a wide array of unguided apps that have come up in the commercial marketplace and there is insufficient information built into the description of apps to help a potential user decide whether a given app might best suit his/her needs [12].

Reviews and meta-analyses of randomized controlled trials of internet-based interventions for depression and anxiety disorders have, in general indicated promising results in term of their efficacy in reducing severity of symptoms [13,14,15,16,17,18]. Despite mounting evidence on efficacy of internet-basedinterventions for common mental health problems, several concerns have been noted by researchers working in this field. Some of these include low uptake of internet-based interventions by individuals who are provided access to the same and high drop-out rates seen in intervention trials. Limited use of active placebo controls in research studies and insufficient evidence about long term gains of internet-based interventions etc. are a few other issues that have been raised [16,19,20]. Mushrooming of un-tested mental health apps, some of which are designed with little involvement of mental health professionals and promoted in the unregulated marketplace has also raised ethical questions [13,21,22].

### 2.2. Rationale for the Study

Although there have been large-scale empirical studies on internet-based interventions for common mental health problems across the globe, most of these are from Western nations and only some of these well-researched apps are freely available to a potential user [23,24,25]. Relatively very little is known about how internet-based methods of delivery of psychological interventions may be received and used in non-Western cultures by individuals for whom they are designed [26,27,28]. A broad scan of literature indicated that although several papers have been published in the last few years on the scope of digital mental health in India, there is a significant dearth of empirical research on internet-based interventions for mental health in the Indian context [29,30,31,32].

The findings of a recently completed National Mental Health survey spanning 12 Indian states has revealed high prevalence of common mental health problems and importantly, the extent of treatment gaps for these problems. As per this survey, one in every 20 persons is estimated to be suffering from major depression. What is more disturbing is the observation that about 85% of those who need professional intervention for depression are not able to access treatment [33]. In this context, an increasing penetration of the internet in India [34] provides an opportunity to examine the potential utility of internet-based interventions for depression in urban Indians.

A review of free depression apps available on Android platforms for Indian users highlighted that most of these apps focus on psycho-education or mood monitoring, and only a few apps incorporate managing suicidal thoughts as a component [25]. This review also indicated that there is limited information built in these apps on their scope and limitations or for educating the users about when to reach out for a higher intensity intervention as well as how to overcome mental barriers related to seeking professional help. The last point becomes especially relevant in a country like India where stigma and negative attitudes towards help seeking make it difficult for individuals to seek professional face-to-face interventions for mental health [35,36]. Self-care apps cannot merely help individuals learn skills to handle their minor mental health issues, but can also include content aimed at strengthening positive attitudes towards seeking professional help whenever it is needed. There is a need for development and testing of internet-based interventions that are culturally appropriate, and address common concerns in clients as seen in clinical practice, while taking in account limited professional resources and low levels of mental health literacy [36,37]. On the whole, the review of research highlighted the need for an internet-based self-help program for depression that covers a wide range of therapeutic components commonly utilized in clinical practice in urban India, and addresses some of the lacunae in free depression apps through including components on psycho-education, managing suicidal thoughts as well as guidance about when to step-up and reach out for professional support. It was in this backdrop that an internet-based self-help program for depression was developed and pilot tested in India. The present paper is aimed at describing the development of the program, as well as the result of the pilot testing of the program. Pilot testing was primarily aimed at documenting patterns of use, perceived gains as reported by the users and short-term outcomes on depressive symptoms, functioning and wellbeing.

## 3. Method

Program development and salient features:Practice and Use Self-help for Depression (PUSH-D) was developed to serve as an internet-based self-care program to help individuals deal with symptoms of depression. The name of the program (hereafter referred to as PUSH-D) was decided following brainstorming within the research team to serve as an easy-to-remember acronym. It aimed at conveying its essence: a self-help program to deal with depression with an emphasis on the role of practice.

### 3.1. Content Considerations

The development of PUSH-D was based on review of publications on internet-based interventions in common mental health conditions, review of depression apps freely available in commercial market place as well as multiple rounds of discussions within the research team. The content development process was guided by the clinical experience of the first two authors who are engaged in providing psychological interventions for adults with varying severity of depression in a tertiary care teaching hospital as well as in a community based well-being center in a metropolitan city of South India. The components were drawn primarily from theoretical frameworks that have been well researched and are commonly applied in clinical practice with urban Indian clients. These included ingredients of cognitive behavior therapy, supportive therapy, interpersonal therapy as well as positive psychology. Thus the coverage attempted to bring the research and practice perspectives together. The other authors provided expertise in public health, psychiatry and use of technology as required during various phases of the project. The following points were taken into consideration in the development of PUSH-D.

At the outset, it was decided that PUSH-D should comprehensively address a wide range of components that are commonly included in face-to-face individual therapies for Indian urban clients with depression. Based on clinical experience in the local culture(apart from empirical literature review), the researchers identified the following components in the initial round of discussion: psycho-education about depression, behavioral activation, learning to identify and correct cognitive errors, worry management, reducing self-criticality and enhancing self-compassion, enhancing self-soothing skills and enhancing an overall sense of mastery. A few additional components were also decided upon, based on the rationale described below.

As prior research indicates high dropouts in internet-based interventions, it was planned to include a component focused on helping users with strategies to keep up their motivation to complete PUSH-D (e.g., scheduling and prioritizing self-care, anticipating gains through practice, self-reinforcement for progress etc.). Our clinical experience as well as research suggests that individuals in a collectivistic culture like India experience barriers to seek help for distress from informal sources due to a wish to avoid placing unnecessary demands on significant others/burden them and the need to maintain harmony [38]. Hence it was decided to include a component focusing on mobilizing support from informal sources for managing distress and generating positive emotions.

Self-care interventions are low-intensity interventions and their users need to recognize when self-help may be insufficient and seeking face-to-face interventions is required. The review of depression apps indicated that several of these do not provide sufficient information to the users regarding the limitations of self-help approach. This information is important for users of self-help apps in a context where stigma and lack of awareness play a major role in non-utilization of services. Hence the last section in the essential zone of PUSH-D aimed to help users become aware as to when professional help seeking might be an appropriate decision. The section also extensively covered various psychological barriers to seeking professional help and ways of overcoming hesitations and ambivalences related to seeking face-to-face interventions in times of need. The above-mentioned components formed the various sections of PUSH-D (Table 1) and were grouped together under the label of Essential Zone.

PUSH-D also contained additional sections put together under the label of optional zone. These sections could be accessed by a user at any point of time depending on need or preference. The optional zone was developed to address additional issues that clients with depression may present with (viz. maintaining sleep hygiene, dealing with interpersonal relationships issues, dealing with grief, and learning relaxation through visualization). A section on dealing with suicidal thoughts was also included, though PUSH-D was not targeted at individuals experiencing significant suicidality and the evaluation process identified and directed individuals with high suicidal risk to appropriate services. This was kept as an optional section thatwas freely accessible to clients at any point. A provision was made in the program to deliverautomatic prompts to clients to go to this section, when their current mood rating indicated high distress along with death-wishes or suicidal thoughts. These mood ratings on a pleasant to unpleasant continuum were captured through the presentation of a single seven-point smiley-based item on each log-in. In addition, the optional zone included sections that provided inputs on maintenance of wellbeing so as to help clients in not just reduction of depressive symptoms but also in enhancing an overall sense of well being over time. The optional zone also contained references and links relevant to the content for users interested in further reading.

A few other points were taken into consideration in the process of development of PUSH-D. All self-care/help strategies introduced were those thatcould be potentially learned by a user without face-to-face interaction with a therapist. These strategies were preceded by a clearly articulated rationale for the same. It was considered important to help users understand why and how certain strategies might help. This rationale was based on available research as well as clinical experience of the authors.

It was ensured that across sections, exercises for practice/assignments for application in daily life were introduced after providing demonstrations/illustrations. Diverse examples from family, social relationships, work and academic domains were used across sections, so as to keep the content relevant to individuals from different backgrounds. An attempt was also made to incorporate examples based on commonly seen themes in clinical practice in the Indian culture (e.g., self-criticality related to one’s role as a parent of an adolescent, responsibilities associated with living together with parents/in-laws) and to avoid examples or wordings that may have low relevance for many Indians (e.g., dating).

### 3.2. Structure and Design Considerations

The salient structural features of PUSH-D as mentioned above included the division of the entire content into essential and optional zones. The users were expected to complete the essential zone section in a predetermined sequence. The sequencing of sections was designed to include the most basic content such as psycho-education and motivation enhancement first, followed by behavioral activation, and then dealing with depressive thoughts. These were considered the basic components to be addressed and hence appeared at the beginning. Subsequent sections were organized so as to build links from one section to another, to the extent feasible. Sequential access to the essential zone sections was planned with the aim of minimizing random browsing and helping users progress through the content in a systematic fashion. Optional zone sections could be accessed by a user at any point of time depending on need or preference.

The program was made available through a webbrowser as well as through a mobile app on the Google Play store so as to provide clients with easy access to the application, as well as allow access in settings with greater privacy. The browser and mobile version were synchronized so that the client could work on a section on one device and seamlessly continue with subsequent activities on a different device.

Since the data recorded by the clients in the app were of a sensitive nature, the communication and storage/access of data had to be secure. HTTPS was used for secure communication to the web services. Access to the program was through a user-ID/password-based login. The client’s personal identifiable information (PII) was maintained separate from the clients’ program data, and all access/updates of such program data was through anonymized identifiers.

### 3.3. Functional Features of PUSH-D

Some of the salient functional features of PUSH-D included the following:

▪ Provision to schedule one’s next session: The users were prompted to plan their next session by filling in the expected date and time while logging out. This resulted in two automatic mobile message prompts on the scheduled date.
▪ Reflections: Space was provided to jot down the summary of what was learnt in each section at the end of the respective sections.
▪ Flagging of sections (“likely to be highly personally relevant for you”): Sections were identified as highly personally relevant for a given user based on pattern of responses to a checklist before the start of Section 1. The items on this checklist corresponded to various section-themes (e.g., for someone endorsing the item on very low level of activity, the behavioral activation section was marked as highly relevant). The sections identified as likely to be very relevant for a user appeared at the end of the checklist and these were also labeled as such when the user reached the beginning of those sections.
▪ Facilitating review: Provision to access one’s responses on various items and a brief section summary through one’s online workbook pages.
▪ Guided navigation through the sections in the essential zone, such that sections could be accessed only in sequential order as mentioned earlier. However, a user could go back to and review an earlier completed section at any time.
▪ Monitoring by the professional: There was a provision for the professional who carried out the initial evaluation to view a given client’s workbook and the session summaries entered by the user.
▪ Facilitating continued application: Provision to create new versions of various templates to practice or to manage different exercises (e.g., weekly monitoring of one’s activities)
▪ Self-monitoring: Provision to examine the time spent per log in and the number of log-ins for self-monitoring
▪ Feedback and reinforcement: Verbal and visual feedback on progress in terms of number of sections and number of exercises completed.

### 3.4. PUSH-D Pilot Trial Method

#### 3.4.1. Design and Sample Selection

The overall PUSH-D trial was meant to examine the socio-demographic and clinical profile of seekers of PUSH-D, pattern of use and short-term outcomes. The present paper is focused on describing patterns of use, short-term outcomes and gains as perceived by the participants. Pattern of use herein broadly refers to the proportion of participants who logged in to the app once or more after being provided access to the app, number of sessions completed, average number of log-ins and duration of log-in time. Short-term outcomes primarily refer to changes in depression and level of functioning as the participants progressed from first log-in to completion of ten sections and two months thereafter. A single group pre-, post- and follow-up design was used, keeping in view of a lack of availability of empirical data on receptivity of such an internet-based self-help program for depression in urban India, as well as the cost and time constraints related to this study.

This being one of the first studies on a mental health app in India, the sample selection criteria were kept minimal. Eligibility criteria for inclusion in the trial were: age18 years and above; educated at least up to high school (ten years of formal education); ability to read and write and understand English; familiarity with basic use of computers and internet; depression severity score of 13 or more on the Beck Depression Inventory (BDI II) [39]. Exclusion criteria were: (a) Those suffering from psychotic disorders/mental retardation/dementia or any other major neurological or psychiatric condition that interfered with the ability to follow instructions and complete the self-help intervention, as determined during the screening; (b) those reporting significant suicidal ideations on any of the twoindices: frequent suicidal thoughts or low sense of control over suicidal thoughts as assessed through items in the basic data sheet and/or moderate to high suicidality score on MINI International Neuropsychiatric Interview (MINI) for current suicidality [40].

**Figure 1:**
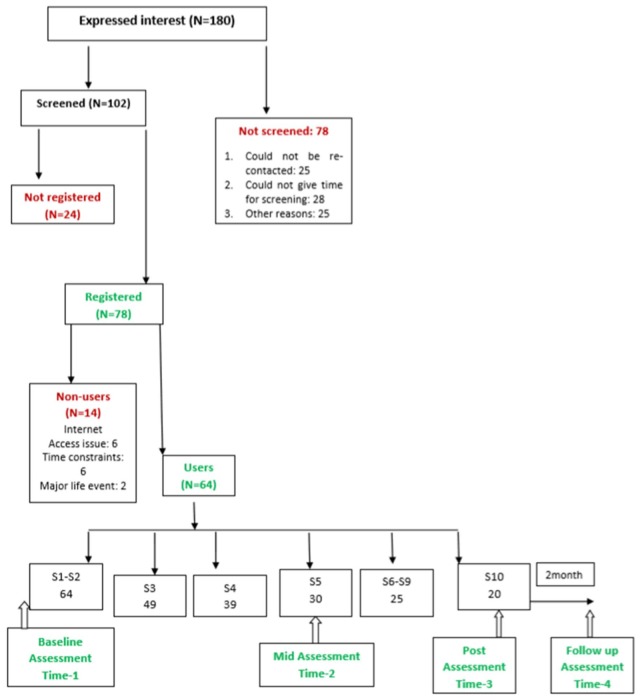
Flow Chart of pilot trial. Note: S1-S10 refer to the 10 sections in the essential zone of PUSH-D & the figures below section numbers refer to number of users who used the respective sections

Those who were clinically adjudged to require face-to-face individual or family or marital therapy for major ongoing stressors (e.g., family discord/complicated grief etc.) were encouraged to seek such interventions at the institute where the study was carried out (or elsewhere as preferred by the individual), instead on being enrolled in PUSH-D trial. This was considered an ethical approach to adopt in view of the researchers’ awareness of the limitations of PUSH-D content to address major life stressors/interpersonal issues in a contextualized fashion. This was decided by the research staff in consultation with the investigators after completion of the initial assessment. The corresponding numbers are mentioned in Figure 1.

The pilot trial was conducted after obtaining approval by the ethics committee of the researchers’ institute. The details regarding announcement of PUSH-D trial and recruitment process as well as basic and clinical profile of those seeking participation in PUSH-D program trial have been described elsewhere in detail [41]. Participation in the pilot testing was solicited through various means. Poster, flyers and write-ups were developed to make the potential participants in the community aware about PUSH-D program and its main features (self-help, internet-based intervention targeting depressive symptoms and utilizing techniques used in psychological interventions for depression in face-to-face sessions). A few core symptoms of depression were presented in the posters, to help people gauge if the distress they were experiencing could indicate possibility of depression. In addition, all the announcements indicated that participation in PUSH-D research trial involved initial screening/evaluation to determine the suitability of PUSH-D program for the given individual and in case it was not found suitable, referral-related guidance would be provided. Announcements were made on multiple offline and online platforms that included posters on notice boards of various local institutions, emails, an announcement on the institute’s website, as well as another website aimed at youth mental health promotion run by the corresponding author, and dissemination of flyers during two health-related exhibitions in the metropolitan city where the researchers are located. Individuals who came across the announcement on PUSH-D and approached the resource team via phone or email were initially provided detailed information about PUSH-D evaluation process, it being a research trial as well as the nature of the PUSH-D program itself.

About two-thirds of the seekers of PUSH-D on whom initial screening and assessments were carried out were treatment naïve and had never sought professional consultation. Reasons for not seeking professional help ranged from lack of awareness about the nature of the problem as well as services, timeconstraints and stigma [32,41].

#### 3.4.2. Assessments

The basic data sheet developed for the present study incorporated sociodemographic details as well as additional details such as major life events in recent past, ongoing stressors, availability of support, self-report of suicidal thoughts if any in the last few weeks, self-perception of the capacity to control suicidal thoughts, past consultation with a mental health professional if any, etc. MINI-International Neuropsychiatric Interview Version 6.0 (MINI) was used to establish psychiatric diagnoses, if any, during the initial evaluation [40]. A Structured Clinical Interview for DSM-5 Personality Disorders (SCID-5 PD) [42], an updated version of the former Structured Clinical Interview for DSM-IV Axis II Personality Disorders (SCID-II) was used for arriving at personality disorder diagnosis, if any. This was also used only during the initial evaluation.

Primary Outcome measures: (a) Depression: current severity of depressive symptoms was assessed using Beck Depression Inventory-II (BDI-II) and Patient Health Questionnaire (PHQ-9) [39,43]. PHQ-9 is shorter in length and hence it was introduced to quickly capture changes at midpoint without increasing respondent burden. (b) Functioning: Self-reported functional impairment was assessed using the Work and Social Adjustment Scale (WSAS) [44]. Higher scores indicate poorer adjustment. In addition a simple clinician-rated simple measure namely, Global Assessment of Functioning (GAF) [45] was also used as an index of functioning. The scores range from 0 (inadequate information) to 100 (superior functioning).

Secondary outcome measures: Rosenberg Self-Esteem Scale [46] and the WHO-FIVE Well-Being Index [47] were used as additional outcomes measures. Higher scores on these measures reflect higher levels of self-esteem and well-being, respectively.

#### 3.4.3. Procedure

Those interested in participating in the initial evaluation/screening process were contacted and evaluation was carried out either in a face-to-face session, Skype session or over telephone, with the consent of the participant.

Face-to-face and Skype sessions were conducted for 45 and 14 participants respectively, while telephonic assessments were carried out for 43 participants. The baseline evaluation was carried out in the first session after obtaining written informed consent. This consent was obtained in the face-to-face sessions, or it was obtained by requesting the participants to send a signed and scanned document over email for those who were interviewed over Skype or telephone.

The baseline evaluation involved listening to the narration of psychological difficulties as described by the interviewees, filling of the basic data sheet, and administration of MINI and SCID-PD, followed by administration of the self-report questionnaires. A brief feedback was provided to the participants regarding the suitability of PUSH-D for their problems or the need for other forms of mental health interventions. User names and passwords were issued if PUSH-D was considered a suitable mode of intervention, while for others, referral-related guidance was provided.

The participants who were registered for PUSH-D were recommended to complete the self-help intervention program within a period of six to eight weeks. It was estimated that a participant would be required to spend on an average of about two hours in a week with the program material (in chunks of 20 to 40 min) over six weeks to complete the entire program. In addition, they were prompted to carry out simple activities and exercises for mood management on a regular basis as is the case in a typical face-to-face intervention for depression. These were built-in exercises across various sections. The users were expected to carry them out/apply the learning between log-ins. A few examples include weekly monitoring of engagement in pleasurable and meaningful activities, monitoring self-critical thoughts and substituting them with self-compassionate thoughts, applying and recording the use of self-soothing techniques when in distress.

Literature suggests that compliance and motivation management are difficult issues in stand-alone self-help programs. The content of the internet-based program was supplemented with periodic telephone assistance, once every 8–12 days as the available research suggests that human contact in some form or the other helps improve adherence and outcomes—these calls were made by research staff members who were trained clinical psychologists. The telephonic assistance was meant to serve as minimal contact/basic assistance, clarify doubts if any, resolve technical issues in navigating, and motivate users to work on the content. The calls were typically brief (lasting for about 15 min) and the timings were fixed as per users’ convenience. These also helped in monitoring severity of distress and addressing any need for referrals to appropriate, face-to-face professional services, if and when needed. Such a provision for referral was made in the research protocol as per the ethical practice. The plan was to exclude data of such participants who might be referred for face-to-face intervention at any point during the trial. However, no participant required such a referral after registration into the PUSH-D program. Periodic mobile prompts with suggestions to maintain efforts at self-care, and short quotes relevant to managing negative emotions and cultivating positive emotions were sent at a frequency of about once in 10–15 days.

Assessments were carried out at four time-points (Table 2). Baseline assessment was carried out at the point of recruitment and soon after screening (T1); midpoint assessment was carried out after completion of 5 out of 10 sections (T2). Post-assessment was carried out after completion of all 10 essential zone sections (T3). Follow-up assessment was carried out twomonths after completion of post-assessment (T4). While the baseline session was conducted face-to-face or via Skype/telephone, the remaining assessments entailed a combination of email and telephone, as per the convenience of the participants.

#### 3.4.4. Analyses

SPSS Version 16.0 was utilized for data analyses. The data entry was checked for errors. Paired sample *t*-tests were used for comparison between two points of time. Repeated measures ANOVA was used for within group comparisons involving more than twomeans. Mauchly’s test was used to examine any significant violation of sphericity assumption. In any instance of violation, degrees of freedom were corrected using the Greenhouse and Geisser estimate [48] to examine significance of Fratio. In cases of a significant F ratio, significant results on post hoc comparisons were made and significant differences between any two timepoints are reported after a Sidak adjustment for multiple comparisons. Changes in proportions were examined using the McNemar test. Effect sizes were calculated in various analyses terms of correlation coefficients as recommended by Field, 2005 [49].

## 4. Results

### 4.1. PUSH-D Trial Sample Characteristics

Following baseline evaluation, 78 participants were registered for PUSH-D while 24 were not registered. Nine out of these 24 participants were not registered due to moderate to high levels of current suicidality. One or more of the following other reasons resulted in exclusion 5 participants each from registration to PUSH-D: lack of comfort with English language, lack of regular access to internet, absence of depressive symptoms and presence of other psychiatric disorders or major family or interpersonal concerns that were adjudged to require face-to-face individual or family therapy (Figure 1).

The basic sociodemographic details of the participants are described in Table 3. An average participant was about 32 years old and both the genders were fairly represented in the sample. Majority of them had a bachelor’s degree and were employed. While about one-third (34.65%) were currently experiencing a major depressive episode, about 65% were experiencing chronic low grade depression qualifying for dysthymia. A comorbid personality disorder (Cluster C) was evident in 6% of the participants. As per the information obtained through the basic data sheet, 18 out of 78 (23%) reported experiencing suicidal thoughts. However 12 of them reported being able to easily manage/control them while 6 reported some difficulty in managing the same.

Major life events in the preceding sixmonths were reported in 46.2% of cases. About 65% reported having never sought mental health consultation any time. On the other hand, 28 (35.9%) reported having consulted a mental health professional for current issues and it was a consultation with a psychiatrist for a majority of them. Out of these 28, 18 were receiving some form of intervention (15 being on stable dose of an antidepressant, one was undergoing counseling and 2 were receiving counseling and antidepressant).

### 4.2. Usage of PUSH-D in Registered Participants

Out of 78 participants who were registered and given PUSH–D log in details, 14 participants (18%) did not use the program or did not complete the first section and were termed nonusers. Thirty out of 64 users (47%) were categorized as partial completers as they completed 5/10 essential sections and provided Time-2 (T-2) repeat assessment data. Twenty out of 64 (31%) participants completed all the ten essential zone sections and provided Time-2 and Time-3 data (T-2 & T-3). Sixteen out of 20 completers additionally provided follow-up assessment data (T1 to T4). Owing to different number of participants available at various points of time, analyses have been carried out separately for partial as well as full completers. These details have been depicted in Figure 1.

The midpoint assessment (T-2) coincided typically with the end of first month after baseline assessment. While, the time interval between post-assessment (T-3) and follow-up assessment (T-4) was fixed for all the participants (2 months), post-assessment was carried out after completion of ten sessions. Except in case of 3 participants, post-assessment was carried out between 6th and 8th weekfor all the others. Three participants needed 12 weeks’ time for completion of ten sessions and providing on post-assessment data. The average interindividual variability in capture of post=assessment data in a majority of instances was of 10 days and was attributable to logistic constraints that came in way for some users to complete ten sections and provide post-assessment data. An average completer logged in 17 times and spent about 30 min on an average per log-in (standard deviation: 14 min). The remaining participants spent about 24 min per log-in on an average (standard deviation: 16 min).

### 4.3. Examination of Outcomes in PUSH-D Users

Mean changes on outcomes at post-assessments: Twenty registered participants completed all the ten essential zone sections and post-assessments. Depression scores decreased significantly from baseline to post-assessment on BDI-II, and also from baseline to midpoint on PHQ-9; while the well-being scores increased in this period. On the other hand there was no significant change evident in self-esteem from baseline to post-assessment. There was a consistent decline on impairment and improvement in global functioning seen from baseline to post-assessment in this subsample (Table 4).

Percentage changes on depression and impairment and shifts in severity in individual participants at post-assessment: Change scores from baseline to post-assessments were also calculated and these were and transformed into change percentages relative to the respective baseline scores. Large mean percentage changes in Beck Depression Inventory scores were noted (Mean: 61.79) followed by those on PHQ-9 (46.25) and impairment (30.98), although variability in individual level gains was evident from large standard deviations.

Using the standard cut offs used for BDI-II, an attempt was made to explore the pattern of shifts in severity of depression in those who completed post-assessment. There was a marked shift in terms of drop in proportion of the sample that was in moderate and severe depression category (from 35% and 40% to 10% each respectively). While 75% of the participants showed a shift to a lower severity category, none showed worsening/shift-to-higher category. The overall shift in severity across categories on BDI-II was significant (McnemarBowker test, *p* < 0.01).

As far as changes in severity of impairment from baseline to post-assessment are concerned, a majority of participants moved from moderately severe impairment to higher levels of functioning. While sixty percent had moderately severe impairment at baseline, twenty percent could be so classified at post-assessment. The overall pattern of shift was significant (McNemar–Bowker test, *p* < 0.05), with 73% of the participants showing a shift to lower impairment category and none showing a worsening.

Thirty participants who completed 5 out of ten essential zone sections were categorized as partial completers. Comparison of scores of the partial completers subsample revealed significant improvements from baseline to T-2 on depression (PHQ-9). Significant decline on impairment was also seen from baseline to T-2 in partial completers (Table 5).

Assessments at midpoint also provided an opportunity to track shift in severity of depression and impairment. There was a drop in the proportion of individuals falling in moderately severe category (PHQ-9) at T2 as compared to baseline (40% at baseline vs. 13.3% at post-assessment). There was a corresponding increase in the number of participants in mild category from 20% to 50%.

Similarly, gains in functioning were seen in partial completers in terms of drop in proportion of participants who were classified as having moderately severe impairment at T2 on WSAS (46.7% vs. 26.7%).

Stability of gains over a period of 2 months following completion of the program could be ascertained in 16 out of 20 completers who could be contacted for the same. Significant gains were made and maintained on depression as measured by BDI-II and PHQ-9, Work and social functioning (decline in impairment) as well as ratings on global assessment of functioning. On BDI-II as well as PHQ-9 measures, depression scores declined significantly from baseline to post-assessment and then further decline was evident at follow-up. PHQ-9 was also assessed at midpoint providing a picture of changes during the program. It was observed that depression scores tended to decline from baseline through midpoint to post- and follow-up assessment resulting in post and follow-up assessment scores being significantly lower than the baseline. Scores on self-esteem and well-being measures improved from baseline to post-assessment. Further, there was a significant improvement in self-esteem and well-being seen at follow-up (Table 6).

Subjective reports of impairment decreased significantly from baseline to post-assessment and this gain was stable at follow-up; the follow-up scores being significantly improved compared to baseline as well as midpoint. As far as clinician ratings of functioning is concerned, there was a similar upward trend seen in functioning, with significant improvement from baseline to post and midpoint to post-assessment, with gains being maintained at follow-up (Table 6).

### 4.4. Supplementary Analyses

Participants who completed 5 sections (i.e., 50% of the program content) and provided repeat assessment data (partial completers, *n* = 30) were compared to those who did not use or discontinued use before completing 5 sections and providing repeat assessment data (*n* = 48, 61.5%). These 2 groups were not significantly different from each other on gender (*p* = 0.48), marital status (*p* = 0.41), educational level (*p* = 0.46), work status (*p* = 0.66) presence of major depression (*p* = 0.32) or dysthymia (*p* = 0.27). Similarly these two groups did not significantly differ from each other on baseline scores on self-esteem, well-being, work and social adjustment, PHQ-9 or BDI –II depression and global assessment of functioning ratings (with all *p*-values being > 0.05). Durations of dysthymia and major depression were not significantly different (*p* = 0.11 *p* = 0.10). On the whole, no baseline variables assessed in the study differentiated between users who completed 5 sessions vs. those who did not. Further supplementary analyses with the subgroup of those who never initiated PUSH-D (after having been provided log in credentials) also revealed similar patterns; with no difference on baseline variables seen, between these participants and the partial completers.

The PUSH-D pilot trial was meant to understand the profile of seekers of such a program in urban India apart from assessing short-term outcomes of its usage. Also, internet-based self-help programs have the potential to be used as supplement to treatment as usual. Hence, no exclusion criteria were used regarding ongoing mental health interventions. As mentioned earlier, 18 of the 78 registered participants reported receiving some form of intervention at the time of baseline assessment. In the sample of completers and partial completers on whom main analyses have been carried out, only 8 participants were undergoing intervention (on stable dosage of antidepressants). Re-running the analysis after exclusion of these 8 participants returned similar results as mentioned above. Significant improvements from baseline to post-assessments were noted on both the measures of depression (BDI: *p* < 0.01, *r* = 0.82; PHQ-9: *p* < 0.05; *r* = 0.68) and on functioning (WSAS: *p* < 0.05; *r* = 0.79; GAF: *p* < 0.05, *r* = 0.93) with large effect sizes.

### 4.5. Reports of Perceived Utility and Feedback from Users

There was a provision at the end of each section for a user to write down a summary of what was learnt in that particular section. This could be revisited/viewed by the user in his/her workbook section. Although all users did not have entries for all the sections; 285 entries were available for content analysis. Most of the content across sections could be classified into four broad categories, namely information, self-awareness, self-motivation, picking a strategy for use/application. Mere repetition of some of the factual content of a section was coded as information gained. Self-awareness was coded for summaries thatexpressed the participants became aware about certain aspects of themselves (e.g., “I realized that I often tend to engage in all-or-none thinking”/“I think that I have been too critical of myself”). Self-motivation was coded wherever the summary content indicated a motivating message that the user wrote for themselves (e.g., “I must keep going, come on, let me just do this”). Picking a strategy was used for putting together those summarieswhich indicated an intention/plan that the user wanted to apply a specific strategy learnt in the section (e.g., “I will make a list of things that are under my control and focus on that first”). It was noted that the largest number of summaries reflected the user picking a specific strategy/plan and reflecting on the same for their use. The second largest category was self-motivating statements/inspirational statements for self, followed by others (Table 7).

At the beginning of each section, the user was provided with a prompt item to reflect on what they had applied/tried to apply based on learning of the previous section. This was a multiple-choice item. Analysis of 279 responses across users and sections on this item revealed that about 50% of responses reflected an intention to use what was learnt; while about 43% of responses indicated that before moving on to the next section, the participants had already used something or the other learnt in the previous section and had found that to be beneficial to varying extent (Table 8).

While hope about new learning and witnessing benefits were seen as the most helpful factors, practice exercises, clarity and comprehensive coverage as well as videos were reported to be the most liked aspects of PUSH–D by the completers. Weekly calls were seen to serve the purpose of feeling less alone and having help available when needed (Table 9).

Emotion management and strengthening a sense of mastery were reported as the most frequent perceived gains by the follow-up participants (Table 10).

Table 11 provides a few excerpts of the encouraging feedback received from the completers of PUSH-D program (*n* = 20).

## 5. Discussion

To the best of our knowledge, PUSH-D is one of the first indigenous internet-based self-care intervention programs in the field of mental health developed by mental health professionals in an academic setting with assistance from a technology partner.

The strengths of the PUSH-D program include a comprehensive coverage of content targeting depressive symptoms and providing opportunities to clients to empower themselves with self-care strategies to deal with depression through use of informative text, videos, exercises, posters and monitoring tools. The content development took into account an exhaustive review of free apps on depression available to Indian users in addition to the existing scientific literature. It was ensured that the scope and limitation of self-care were emphasized adequately and a section to educate users about when to seek professional help and how to break mental barriers to seeking such help was included as part of the essential zone. Mobile prompts and basic telephonic assistance were added to enhance chances of completion of PUSH-D, in view of available literature indicating poor rates of completion of unassisted internet-based mental health programs.

A typical PUSH-D trial participant was a working young adult of either gender, with at least three years of college education. Most participants were suffering from major depression or dysthymia and only a small proportion had comorbid psychiatric condition (mostly anxiety disorder or cluster C personality disorder). Most participants had moderate to severe depression and significant impairment in functioning at baseline. Almost two-thirds of the participants had never sought mental health consultation at any point in the past.

Slightly less than one-fifth of the registered participants were nonusers. A somewhat larger proportion of registered participants (50%) completed 40% of the sections though they did not complete 50% of the essential zone or provide repeat assessment (Time-2) data. No baseline variables differentiated between those who did not complete five sections vs. partial completers. The former group was also difficult to connect to and engage with through weekly telephonic calls. This pattern somewhat mirrors observations in other studies, indicating that a significant proportion of individuals do not complete internet-based mental health self-help programs. The proportion of nonusers in the present study was lower (17.9%) than that reported earlier. Out of the 420 participants in a study involving two active app based interventions, 58% did not download the apps, despite having gone through eligibility screens, giving consent and completing baseline evaluation. Moreover, adherences to both the apps declined over time [26]. Decrease in user engagement over time has been reported in several studies [16].

The available literature reviewed earlier indicates that user-uptake and retention are major challenges related to self-care programs. There is a need for further studies to enhance understanding of modifiable factors related to non-use of self-help intervention like PUSH-D, despite initial inclination, expressed interest and investment of time to undergo the evaluation process. Provision of feedback on progress/summary of progress built into depression apps may have some utility [16] and this feature was incorporated in PUSH-D. It has been observed that engagement with the app may be a very important variable, but it is often likely to be less than optimum due to a variety of reasons such as life-roles and responsibilities in addition to technical issues such as wi-fi access etc. [13]. Clients with low treatment motivation may benefit from approaches aimed at motivational enhancement [23]. An initial section of PUSH-D incorporated inputs to sustain motivation to complete PUSH-D program. In previous studies, adherence to internet-based interventions is reported in terms of indicators such as number of log-ins, duration of exposure to intervention, number of modules or exercises completed, and number of postings etc. [50]. A review indicated that about 50–70% of the internet-based intervention modules for depression are completed by participants of research trials [51]. Reviews also indicate that studies need to report as to how much of the internet-basedself-care tool is completed by participants who do not complete the entire tool that is offered [52]. In the present study, 47% of the users completed 50% of the sections (partial completers) and showed significant improvements on primary outcome measures. About 39% of the users completed 90% of the sections (Figure 1). These data suggest adherence rates to be somewhat similar to other studies in about half of the users. Most completers showed sustained engagement in the program as evinced by consistent progress from one section to another, amount of time invested per log-in, number of log-ins and ease of connecting through the weekly telephonic calls. Based on average number of log-ins and average duration per log in, as mentioned earlier, it is estimated that an average completer spent 8.5 h going over the entire program content. Qualitative data presented earlier also suggested fair engagement with the strategies suggested in various sessions. It was difficult to reach out to participants who did not complete 10 sections after Time-2, though attempts were made to connect over phone/mail. Information available from some of these participants suggested the role of factors such as major life event, work demands, vacation, and physical ill health or lowered felt need resulting in dis-continuation/non-completion.

The examination of outcomes highlights the potential utility of PUSH-D in individuals with depression. The completers’ data suggests significant and consistent decline in depressive symptoms during the PUSH-D program and maintenance of gains at 2-month follow-up. Mean percentage changes at post-assessment as compared to baseline severity were calculated. This analysis indicated large average gains on BDI-II (62%). In a previous study, a reduction of 17.5% on BDI-II, was reported to be minimal clinically important difference (MCID) while the corresponding estimate for individuals with longer duration depression (who were non-responders to antidepressants) was noted to be higher at 32% [53]. The shifts in severity of depression were examined as another approach to establish the clinical meaningfulness of statistically significant changes in the present study. Majority of the participants with baseline levels of moderate to severe depression moved up to minimal/mild depression by post-assessment. Similarly, functional impairment reduced significantly in completers as per self-report as well as clinical ratings. Moreover a majority of the participants moved from moderately severe impairment to higher levels of functioning at post-assessment.

The partial completers’ sample provided another opportunity to examine changes in depression and impairment in functioning after completion of 50% of PUSH-D program. This analysis too showed a significant decline in depression, improvement in functioning as well as a drop in proportion of partial completers falling in severe/moderately severe categories (depression and impairment) at Time-2 as compared to baseline. The analysis with this sample suggests that completion of all the essential zone sections are perhaps not required for significant positive changes in depressive symptoms and improvements in functioning to manifest. This seems understandable as the first five sections of PUSH-D contained a few major ingredients of the self-care intervention program namely, psycho-education, improving self-care motivation, behavioral activation and dealing with depressive cognitions apart from managing worries. Along somewhat similar lines, results of a clinical trial mentioned earlier [26] showed that 45–46% of the users manifested at least 50% reduction in depression severity by 4 weeks. This trial also highlighted that most did not use the apps as instructed and this was the case despite differences between apps in terms of content and user experience. This finding is similar to the present study observations on user engagement.

Secondary outcomes were assessed in terms of self-esteem and well-being. On both these measures, there was steady improvement in scores with the overall difference between time-points being significant at post-assessment on well-being. Significant changes in these two variables were evident from post-assessment to follow-up period. The overall pattern suggests that changes on well-being and self-esteem are likely to manifest after significant decrease in depressive symptoms and improvement in functioning. This finding raises the hypothesis that apps for depression may impact on indices of well-being, beyond reduction of depressed mood and improvement in functioning.

Examination of effect sizes provided an estimate of magnitude of effects. Moderate effect sizes in terms of reductions in depressive symptoms have been noted in CBT based online interventions, but a meta-analysis indicated low impact on functioning [10,54]. In the present study sample, large effect sizes were noted in all analyses involving changes in BDI-II scores at various time points and medium to large effect sizes were noted on PHQ-9. Similarly moderate to large effect sizes were noted on most analyses of changes in level of impairment in functioning as well as on secondary outcomes.

Summaries entered by the users at the end of various sections and user feedback at post/follow-up assessments provided crude pointers to factors that might have contributed to gains observed. These include finding and picking up a useful strategy, enhanced self-awareness and improved motivation to engage in self-care. The analysis of brief responses to an item at the start of various sections indicated that about 50% of responses reflected an intention to use what was learnt; while about 43% indicated that, the participants had already used something or the other learnt in the previous section and had found that to be beneficial to varying extents. The feedback received at follow-up revealed that enhanced emotion regulation efficacy and enhanced overall sense of mastery emerged as most frequently experienced gains, in addition to better self-awareness and a sense of acceptance, improved social connectedness and goal striving. These are likely to have played a role in enhanced self-esteem and wellbeing as documented at follow-up. Moderate to high effect sizes observed for almost all the outcome variables in the present sample may have been contributed by several factors such as (a) a large proportion having significant distress to begin with; (b) having had no prior exposure to counseling/therapy; (c) comprehensiveness of content-coverage and (d) availability of basic telephonic support.

### Limitations and Future Directions

The study has several limitations such as small sample sizes at various time-points, lack of a control group to tease out the effects of passage of time and a short follow-up. Variability in post-assessment time-points (between 6–8 weeks for most users) is another limitation of the study. Replication of findings in randomized controlled trials and longer term follow-up can help in more firmly establishing the efficacy of the PUSH-D program Comparison with a control group (e.g., a group undergoing pharmacotherapy for depression) may be helpful in assessing the robustness of the findings. Low rates of program completion raise concerns that need to be addressed. Simplification in the design of one or two initial sections of the app that involve multiple exercises as well as allowing users to self-select and access sections that are most personally relevant are some of the modifications that are being currently considered. The utility of such design changes for user-retention would require further study. In view of other studies that describe similar challenges in user-retention, it may be helpful to examine the role of self-regulation deficits in non-completion of internet-based self-help programs [55].

In a country like India with multiple languages as well as the likely confound between knowledge of English and educational levels, PUSH-D‘s utility is most likely limited to individuals in the middle and upper socioeconomic strata, with some years of college education, internet access and comfort with English.

Relatively higher levels of anonymity and privacy, flexibility of usage, ease of access, low delivery costs and standardized content have been cited as some of the advantages of internet-based mental health interventions [1]. PUSH-D was indigenously designed to address issues such as (a) lack of freely available internet-based interactive self-care tool for urban Indians dealing with depression; (b) inadequate coverage of therapeutic components in existing apps to suit a variety of client needs; and (c) lack of emphasis on enhancing self-care motivation, managing suicidal thoughts, maintaining well-being and educating about when to step-up. Research from the Indian subcontinent indicates a very high treatment gap for depression with a very small proportion of individuals needing professional intervention receiving the same [33]. Notwithstanding the limitations of the present study, programs such as PUSH-D can improve self-awareness and empower users with self-care skills and bypass time and cost constraints related to seeking face-to-face psychological interventions for depression. Most of the PUSH-D participants had never availed professional services in the past and several had moderate-to-severe levels of depressive symptoms. Completion of PUSH-D, including partial completion (50% of PUSH-D sections) was associated with statistically and clinically significant gains of moderate to large effect size on depression and functioning. These gains were stable over a two-month follow-up. Additional data from the participants in terms of intention as well as application of what was learned to varying extent, average time spent on the program and self-reported gains are encouraging. Even when applicable or utilized by only a segment of the population, it still has a potential to reducing overall treatment gap for depression in acountry with a scarcity of trained professionals, as well as low rates of people seeking professional help due to factors such as stigma [32,41,56]. The PUSH-D trial examined its potential use as a first line of management for depression and dysthymia in line with a stepped-care approach. Its utility for individuals with significant co-morbid psychiatric disorders (e.g., cluster B personality disorders, substance use disorders and OCD etc.) can be examined. Its additive value as a supplementary program in routine psychiatric outpatient settings as well as for bolstering and maintaining gains in those undergoing face-to-face psychological interventions individual therapy are also useful issues for exploration in further studies.

## 6. Conclusions

The limitations notwithstanding, the overall findings suggest that internet-based self-help program PUSH-D has potential appeal and utility for urban Indians, especially those suffering from mild-to-moderate severity of depression, who have not sought professional consultation for their mental health concerns. Further research on PUSH-D, the use of multipronged strategies to ensure uptake (e.g., simplification of design to reduce attrition, availability in the national language, i.e., Hindi, and enhanced public awareness) along with large-scale dissemination can provide an important avenue to reduce the treatment gap for depression in India.

## Figures and Tables

**Table 1:** Content of PUSH-D.

Essential Zone		
Section Number	Section Name	Brief Description
1	Understand depression	Checking one’s beliefs about depression, busting myths and recognizing depressive symptoms
2	Enhance self-care motivation	Anticipating motivational blocks to complete the program & empowering self with strategies to keep up the motivation
3	Activate	Learning about utility of behavioral activation, systematically planning and monitoring one’s engagements
4	Deal with depressive thoughts	Learning to recognize thinking-errors common in depression and substituting unhelpful thoughts with more helpful ones
5	Managing excessive worries	Learning to handle excessive worries, learning the concept of wise mind
6	Learn self-compassion	Recognizing the problems with self-criticality, the utility of self-compassion and working on ways to cultivate a self-compassionate stance
7	Strengthen self-soothing skills	Identifying the use of healthy self-soothing practices for use in order to deal with intense negative emotions
8	Regain a sense of mastery	Managing the sense of feeling overwhelmed by multiple problems and regaining a sense of mastery
9	Harness the power of support	Identifying and overcoming mental barriers to seeking support for managing depression
10	Step-up	Learning to recognize when self-help alone is insufficient and managing psychological barriers to reaching out for professional help for depression

**Table 2:** Measures used during the pilot trial of PUSH-D.

Measures	Baseline (T1)	Midpoint (T1) after Completion of 5/10 Sections of PUSH-D	Post (T3) after Completion of 10/10 Sections of PUSH-D	Follow-up (T4) 2 Months after Post-Assessment
Depression BDI-II	✓	-----	✓	✓
Depression PHQ-9	✓	✓	✓	✓
Self-esteem (Rosenberg Self-Esteem Scale)	✓	------	✓	✓
Well-being Index WHO-5	✓	------	✓	✓
Functional Impairment (Work & Social Adjustment scale)	✓	✓	✓	✓
Global Assessment of Functioning	✓	✓	✓	✓

**Table 3:** PUSH-D trial sample characteristics-I (*n* = 78).

Variables	Frequency	Percentage
**Gender**		
Male	40	51.3
Female	38	48.7
**AGE Mean (SD) 32.28 (12.98)**		
17–25	24	25.6
26–35	32	41.03
36–50	17	21.80
Above 50	5	6.41
**Region**		
Bangalore	55	70.5
Outside Bangalore but within Karnataka	3	3.8
Other states in India	20	25.6
**Education**		
Under Graduation	11	14.1
Graduation	36	46.2
Post-Graduation	31	39.7
**Religion**		
Hindu	67	85.9
Muslim	8	10.3
Christian	2	2.6
Others	1	1.3
**Marital Status**		
Single	46	59.0
Married	30	38.5
Separated	2	2.6
**Work Status**		
Job seekers/currently unemployed	18	23.1
Employed	38	48.7
Student	17	21.8
Retired	5	6.4
**Living Arrangement**		
Hostel/Paying Guest	25	32.1
With Family	53	67.9
**Individual Income in INR (Per Month)**		
Up to 20,000	4	5.1
20,001–30,000	5	6.4
30,001 and above	42	53.8
Do not Wish to Answer	27	34.6

**Table 4:** Changes from baseline (T1) to post-assessment (T3) in completers (*n* = 20).

Variables	Mean (SD)					Effect Size (r)
	T1	T 2	T3	F/*t* Value	Post hoc Differences	
Depression (BDI)	26.16(8.89)	-	10.53 (10.47)	7.22 ***	-	0.86
PHQ-9	10.83 (5.67)	7.06 (5.53)	5.22 (4.80)	10.83 **	2 < 1, 3 < 1	0.54 (1–2)
						0.42 (2–3)
Self-Esteem	25.82 (4.81)	-	28.06 (5.18)	1.84		0.40
Well-being	10.67 (6.18)	-	15.06 (5.93)	3.08 ***	-	0.59
Impairment	21.10 (8.41)	15.74 (10.74)	12.37(8.99)	12.59 **	3 < 1	0.51 (1–2)
						0.47 (2–3)
Global Functioning (GAF)	71.84 (6.91)	73.68 (6.63)	79.84 (5.42)	75.50 **	2 > 1, 3 > 1, 3 > 2	0.54 (1–2)
						0.91 (2–3)

** *p* < 0.01, *** *p* < 0.001.

**Table 5:** Changes in partial completers on select variables (*n* = 30).

Variables	Mean (SD)			
	T1	T2	*t* Value	Effect Size (r)
Depression (PHQ-9)	9.86 (5.79)	7.14 (5.64)	2.84 **	0.47
Impairment (WSAS)	20.00 (8.63)	14.66 (9.78)	3.44 **	0.54

** *p* < 0.01.

**Table 6:** Changes from baseline and stability of gains in follow-up sample (*n* = 16).

Variables	Mean (Standard Error)				F	Significant Differences on Post hoc Analyses	Effect Size (r)
	Time-1	Time-2	Time-3	Time-4			
BDI-Depression	25.44 (2.34)	-	9.94 (2.32)	6.25 (1.84)	56.51 ***	3 < 1, 4 < 1,4 < 3	0.90 (1–2)
							0.59 (2–3)
PHQ Depression	10.46 (1.56)	6.93 (1.30)	5.27 (1.22)	3.20 (1.20)	14.33 ***	4 < 1, 3 < 1, 4 < 3	0.61 (1–2)
							0.37 (2–3)
							0.65 (3–4)
Self-esteem	25.71 (1.06)	-	27.64 (1.31)	30.14 (1.42)	9.65 ***	4 > 1, 4 > 3	0.43 (1–2)
							0.67 (2–3)
Well-being	14.80 (1.48)		18.67 (1.41)	20.87 (1.50)	8.68 ** (corrected)	4 > 1, 4 > 3	0.56 (1–2)
							0.60 (2–3)
Impairment (WASA)	20.69 (2.24)	15.50 (2.31)	12.69 (2.13)	11.25 (2.34)	11.96 ***	3 < 1, 4 < 1	0.54 (1–2)
							0.41 (2–3)
							0.27 (3–4)
GAF	70.50 (2.03)	72.00 (2.26)	79.20 (1.82)	81.00 (2.21)	40.86 *** (corrected)	3 > 1, 4 > 1, 3 > 2 4 > 2	0.54 (1–2)
							0.93 (2–3)
							0.46 (3–4)

** *p* < 0.01, *** *p* < 0.001.

**Table 7:** Emergent themes in session summaries provided by participants. (Number of summaries = 285).

Sections (1–10)	Emergent Themes in Summaries					
	Brief Mention of Information Gained	Self-Awareness	Self-Motivation	Picking a Strategy for Use	Others	Total
S1	30	14	4	-	Feeling that one is not alone-4 General comments: 5	**57**
S2		1	15	15	General comments: 14	**45**
S3		2	5	30	General comments: 2	**39**
S4		8	1	17		**26**
S5	1	5	1	18		**25**
S6		3	4	11		**18**
S7			2	17	Self acceptance-2	**21**
S8			1	18		**19**
S9		3	7	8		**18**
S10		2	7	1	General comments: 7	**17**
**Total Frequency (Percentage)**	**31 (10.88%)**	**38 (13.33%)**	**47 (16.49%)**	**135 (47.37%)**	**34 (12.0%)**	**285**

**Table 8:** Reflection at the beginning of a new section on utilization of what was learnt in the previous section.

User Reflections (*n* = 279)									
Did not Use		Tried Using		Will Try to Use		Used and Found Somewhat Useful		Used and Found Quite Useful	
Frequency	%	Frequency	%	Frequency	%	Frequency	%	Frequency	%
4	1.4	17	6.09	138	49.46	48	17.20	72	25.81

**Table 9:** Helpful factors and suggestions as perceived by the completers.

Helpful Factors/Suggestions	Theme	Description	Frequency	Percentage
Helpful factors for completing the program (*n* = 20)	Professional support	Support by the PUSH-D team	4	20
	Curiosity to uncover and learn	Sequential structure of the program	2	10
	Witnessing the benefit	Feeling better by using the program	5	25
	Hope about new learning to reduce distress	Hoping to feel better by using PUSH-D and learning new strategies to feel better	7	35
	Credibility	PUSH-D developed by a premier mental health institute	2	10
Most liked aspects of the program (*n* = 35)	Posters	Informative posters within various sections	3	8.57
	Self-help	PUSH-D as a source of learning to help oneself	2	5.71
	Videos	Videos used in various sections	5	14.29
	Demonstrations	Demo-exercises before practice exercises	3	8.57
	Structure	Sequential structure of program and step-by step content within sections	3	8.57
	Practice Exercises	Ready-to-use forms for applying what is learned	10	28.57
	Reminders	Reminders before start of the next section—about applying what was learnt and feedback to monitor progress	3	8.57
	Clarity of content and comprehensive coverage of different themes across sections	New content in every section, clear content	6	17.4
Suggestions for change (*n* = 11)	Add humor	Increase content with humor	2	18.18
	Add more images	More posters	1	9.09
	Add music	Light music in the background	1	9.09
	Reduce textual content and increase activities	More practice points	5	45.45
	Provision for Feedback from user to admin	Feedback from user to admin after every activities	1	9.09
	Improve ease of continuity	Ease of refreshing/going back to previous stage for those logging after a gap	1	9.09
Weekly Calls: Utility (*n* = 19)	Reminder	To continue PUSH-D	5	26.32
	Motivation/support	Someone available to help/support and care for me	8	42.11
	Guidance	Provide clarity/guidance when required	6	31.58
Any technical issues/challenges (*n* = 6)	Navigation	Difficulties in moving forward (initial trial users)	4	-
	Video	Videos not playing	2	-

**Table 10:** Self-perceived gains (*n* = 25) from learning based on PUSH-D content (Follow-up assessment).

Broad Theme	Description	Frequency *	Percentage
Emotion management	Managing worries, Controlling anger, Identifying and dealing with negative thoughts	6	24
Improved social connection	Decreasing social withdrawal	3	12
Improved self awareness	Better aware of one’s strengths and weaknesses	3	12
Enhanced sense of self acceptance and compassion towards self and others	Reduced self-criticality, increased tolerance	3	12
Enhanced sense of mastery	Improved focus on controllable factors in life, feeling confident about dealing with issues	5	20
Goal directedness	Being focused and persistent about one’s goals	3	12
Optimism	Looking forward to future	2	8

* More than one category of gains was reported by the follow-up participants.

**Table 11:** Excerpts of general feedback by users (verbatim).

❖ Availability of PUSH-D itself made me feel that there is something to help me. This thought was itself motivating. ❖ The use of activities/exercises and other contents from PUSH-D were very interesting and it has made a huge impact on me. ❖ PUSH-D is very helpful for working people like me who can’t take out time to go to a mental health professional. ❖ The module was very engaging and insightful. ❖ It was very convenient to use. It will be useful to people who are not able to share their problems with others. ❖ The division of the material into knowledge point, toolbox, practice point, commitment etc. aids in clarity and the concepts just seep in. ❖ It helped me to understand that I have ways to fight my depression without medications. ❖ It is very diverse and dynamic. It has colorful charts, posters, videos, diagrams and scales. That breaks the monotony and encourages engagement. ❖ It gives a record of how ‘I am doing’. How much time I have spent on the website, how many exercises I have completed, how far I have to go.

## Notes

**Funding:** The authors gratefully acknowledge funding for this research by the Indian Council of Medical Research (ICMR), New Delhi, India.
